# Femoral neck shaft angle in relation to the location of femoral stress fracture in young military recruits: femoral head versus femoral neck stress fracture

**DOI:** 10.1007/s00256-020-03661-z

**Published:** 2020-11-03

**Authors:** Dong-Kyu Kim, Tae Ho Kim

**Affiliations:** Department of Radiology, The Armed Forces Capital Hospital, 81 Saemaeul-ro 177 beon-gil, Bundang-gu, Seongnam, 13574 South Korea

**Keywords:** Femoral neck shaft angle, Femoral head stress fracture, Coxa Valga, Coxa vara

## Abstract

**Objective:**

To evaluate the influences of the femoral neck shaft angle (FNSA) on the location of the femoral stress fracture and to assess the potential differences in FNSA between fractured and normal femurs.

**Materials and methods:**

Thirty-seven patients with femoral stress fractures who underwent both plain hip radiographs and MRI, from January 2016 to September 2019, were retrospectively included. Patients were classified as having either femoral head stress fracture (group A, *n* = 26) or femoral neck stress fracture (group B, *n* = 11). The FNSA was measured in anteroposterior (AP) hip radiograph. The Mann-Whitney *U* testing was used to compare the continuous values between the two groups. A receiver operating characteristic (ROC) analysis was used to evaluate the value of FNSA for predicting the risk of femoral stress fracture.

**Results:**

The FNSA was significantly higher in group A (median 135.9°, range 129.5–138.6°) than group B (median 124.3°, range 119.5–129.0°) (*p* < 0.001), but there were no significant differences in other clinical factors. Furthermore, the FNSA was significantly higher at the fractured femurs (median 135.9°, range 129.9–138.6°) than contralateral normal femurs (median 127.9°, range 123.8–132.1°) in the patients with unilateral femoral head stress fracture (*n* = 22) (*p* < 0.001). The ROC analysis revealed that the area under curve (AUC), sensitivity, and specificity for predicting the risk of femoral head stress fracture were 0.807, 72.7%, and 68.2%, respectively, at a FNSA cutoff of 131.0°.

**Conclusion:**

FNSA was associated with the location of the femoral stress fracture. In addition, FNSA could serve as a predictive factor for the risk of femoral head stress fractures.

## Introduction

Femoral stress fractures occur in individuals of all ages but can particularly impact young individuals who engage in physical activities such as running. This is because pathophysiologically lower extremity bone stress injury is a type of overuse injury [1–3]. There are known extrinsic and intrinsic factors (e.g., prior stress fracture, training intensity, smoking, and metabolic disease) associated with an increased risks of femoral stress fractures [4, 5]. While military recruits, who consist of healthy young men, are controlled for most of the known risk factors for stress fracture including chronic renal disease, metabolic bone disease, and bone tumors, military recruits are among the most suffered groups from femoral stress fractures owing to abrupt increases in the training intensities [3, 5, 6].

Other than those extrinsic and intrinsic factors associated with stress fractures, some previous studies reported that hip geometry could also serve as an intrinsic factor related to femoral stress fractures [7, 8]. Altered femoral neck geometry is classified into either coxa vara or coxa valga, according to the femoral neck shaft angle (FNSA). Coxa vara is defined when the FNSA is less than 120° and coxa valga is when the FNSA is greater than 135° [9]. Previous studies suggested that coxa vara could be a risk factor for femoral neck stress fracture while coxa valga could be a risk factor for knee joint osteoarthritis [10, 11]. However, studies that evaluate the relationship between coxa valga and femoral stress fracture are lacking. In addition, to our knowledge, no prior studies have determined the location (i.e., head versus neck) of the stress fracture in the femur depending on the FNSA.

Therefore, the purpose of our study is to evaluate the influence of FNSA on the location of the femoral stress fracture, given that differences in FNSA could affect the induced stress on the femur [2]. Furthermore, we aimed to assess the potential difference in FNSA between the fractured and the contralateral normal femurs in the same patients.

## Materials and methods

### Study population

This retrospective study was approved by our institutional review board and requirements for informed consent were waived.

Patients who underwent hip magnetic resonance imaging (MRI) due to hip pain from January 2016 to September 2019 (*n* = 1346) in a military tertiary care hospital were identified. Among them, patients diagnosed with a femoral stress fracture (*n* = 37) based on hip MRI were included in this study. Patients enrolled in this study were divided into one of two groups: those with a femoral head stress fracture (group A) and those with a femoral neck stress fracture (group B) based on the retrospective review of hip MRI examinations using our picture archiving and communication system (PACS).

### MRI protocol

MRI examinations were performed using 1.5-T (Signa Explorer, GE Healthcare, USA) or 3.0-T (Discovery MR 750w, GE Healthcare, U.S.A.) MR scanners with the unenhanced hip protocol. The standard protocol of 1.5-T MRI consisted of axial T1-weighted (repetition time (TR)/echo time (TE) 632/9 ms, 4-mm slice thickness, 0.5-mm gap), axial and coronal T2-weighted (TR/TE 3102/77 ms, 4-mm slice thickness, 0.5-mm gap), and T2-weighted fat saturation (TR/TE 4500/77 ms, 4-mm slice thickness, 0.5-mm gap) sequences through the entire pelvis using a 32-channel body coil. The field of view (FOV) for each sequence was 42 × 42 cm with a 384 × 256 matrix. The protocol of 3.0-T MRI consisted of axial T1-weighted (TR/TE 455/8 ms, 4-mm slice thickness, 0.5-mm gap), axial and coronal T2-weighted with fat saturation (TR/TE 4287/81 ms, 4-mm slice thickness, 0.5-mm gap), and coronal short tau inversion recovery (STIR) sequence (TR/TE 8291/47 ms, inversion time 150 ms, 4-mm slice thickness, 0.5-mm gap) using a 23-channel body coil with a FOV of 41 × 41 cm and 416 × 288 acquisition matrix.

### Image analysis

Plain radiographs were obtained for each patient on the same day of the MRI examination from the ZeTTA PACS Viewer 2001 (Taeyoung Soft, Korea). Standing anteroposterior (AP), frog-leg, and lateral views of both hip joints were obtained from all patients. To assess the FNSA values of all patients, plain radiographs of standing hip AP views were retrospectively and independently reviewed by two radiologists with 5 and 10 years of radiology experience, respectively, who were blinded to any clinical patient information (i.e., side (right vs. left) of fractured femurs or locations of stress fractures). The FNSA was measured as the angle between the axis of the femoral neck and shaft [5, 12]. The FNSA classification was achieved by classifying femurs with FNSA less than 120° into the coxa vara and femurs with FNSA greater than 135° into the coxa valga. The others were classified as normal [9].

Hip MRI was also retrospectively and independently reviewed by two radiologists. To minimize recall bias, there was at least a 2-week interval period between the interpretations of plain radiographs and MRI. Bone marrow edema was defined as ill-defined high signal intensity area on T2-weighted fat saturation or STIR images. Patients were then diagnosed with femoral stress fractures if there was bone marrow edema with a noticeable fracture line in the femur. Reviewers were asked to indicate the sides of the fractured femurs and locations of the stress fractures.

### Clinical records

To investigate clinical factors that could affect femoral stress fracture, age, body mass index (BMI), follow-up period, location of hip pain (anterior, posterior, medial, or lateral sides), worsening of pain after training activity, interval periods from the clinical visit to MRI examination, and smoking history were collected from the electronic medical charts. Subjective pain was evaluated with the use of the numeric rating scale (NRS) at the time of the clinical visit.

### Statistical analysis

All statistical analyses were performed with SPSS 25.0 for Windows (SPSS Inc., IBM, Armonk, NY, USA). Data were tabulated as the median with interquartile range (IQR) for continuous variables, and as absolute numbers with percentages for categorical variables. The continuous variables of the two groups were compared using the Mann-Whitney *U* test, while categorical variables were compared using chi-squared or Fisher’s exact tests. *p* values < 0.05 were considered statistically significant. Interobserver agreement regarding image analysis was evaluated by kappa (κ) statistics for categorical value (i.e., FNSA classification) and intraclass correlation coefficients (ICCs) for continuous value (i.e., FNSA). Kappa values were indicated as follows: less than 0.20, poor agreement; 0.21–0.40, fair agreement; 0.41–0.60, moderate agreement; 0.61–0.80, good agreement; and greater than 0.81, excellent agreement. ICC results were interpreted according to the following criteria: poor (ICC < 0.50), moderate (0.50 < ICC < 0.75), good (0.75 < ICC < 0.90), and excellent (ICC > 0.90). [13, 14]. A receiver operating characteristic (ROC) analysis was conducted to assess the performance of FNSA for the prediction of the risk of femoral stress fracture, based on the values of sensitivity, specificity, and area under curve (AUC). The optimal cut-off value was determined to maximize the sum of sensitivity and specificity.

## Results

### Baseline characteristics

The baseline characteristics of the study population are summarized in Table 1. Our study population consisted of 37 men, with a median age of 20.0 years (IQR: 20.0–21.0 years). Of these 37 patients, 26 patients with femoral head stress fractures (bilateral in four patients) were assigned to group A (median age 20.0 years, IQR 20.0–21.0 years) and 11 patients with femoral neck stress fractures were assigned to group B (median age 20.0 years, IQR 20.0–22.3 years). All the included patients suffered from anterior hip pain and worsening of pain after training activity. There were no significant differences in age, BMI, follow-up period, interval periods from clinical visits to MRI examination, and NRS between the two groups.

**Table 1:** Baseline characteristics of the study population

	Group A	Group B	*p* value	Total
Age (years)*	20.0 (20.0–21.0)	20.0 (20.0–22.3)	0.807	20.0 (20.0–21.0)
BMI (kg/m^2^)	21.5 (19.9–23.9)	22.2 (20.1–27.7)	0.514	21.5 (20.1–24.5)
F/U periods (months)	7.7 (5.4–15.9)	13.2 (6.2–16.7)	0.997	9.4 (5.4–16.2)
Time interval (days)**	10.4 (7.1–13.2)	10.2 (7.2–13.0)	0.891	10.3 (7.1–13.2)
Smoking, *n* (%)			0.864	
Non-smoker	17 (65.4)	7 (63.6)		24 (64.9)
Ex-smoker	3 (11.5)	2 (18.2)		5 (13.5)
Current-smoker	6 (23.1)	2 (18.2)		8 (21.6)
NRS	2.5 (2.0–3.0)	2.0 (2.0–3.0)	0.512	2.0 (2.0–3.0)
FNSA (°)	135.9 (129.5–138.6)	124.3 (119.5–129.0)	< 0.001	130.5 (127.4–137.1)
FNSA classification, *n* (%)			0.004	
Normal	16 (53.3)	9 (81.8)		25 (61.0)
Coxa vara	0 (0.0)	2 (18.2)		2 (4.9)
Coxa valga	14 (46.7)	0 (0.0)		14 (34.1)

Group A: 26 patients with 30 femoral head fractures (bilateral in 4 cases), Group B: 11 patients with 11 femoral neck fractures

*BMI*, body mass index; *F/U*, follow-up; *NRS*, numeral rating scale; *FNSA*, femoral neck shaft angle

*Results of continuous values are expressed as the median with interquartile range (25–75%)

**Time interval: interval periods from the clinical visit to MRI examination

### FNSA differences according to the location of the femoral stress fractures

**Fig. 1:**
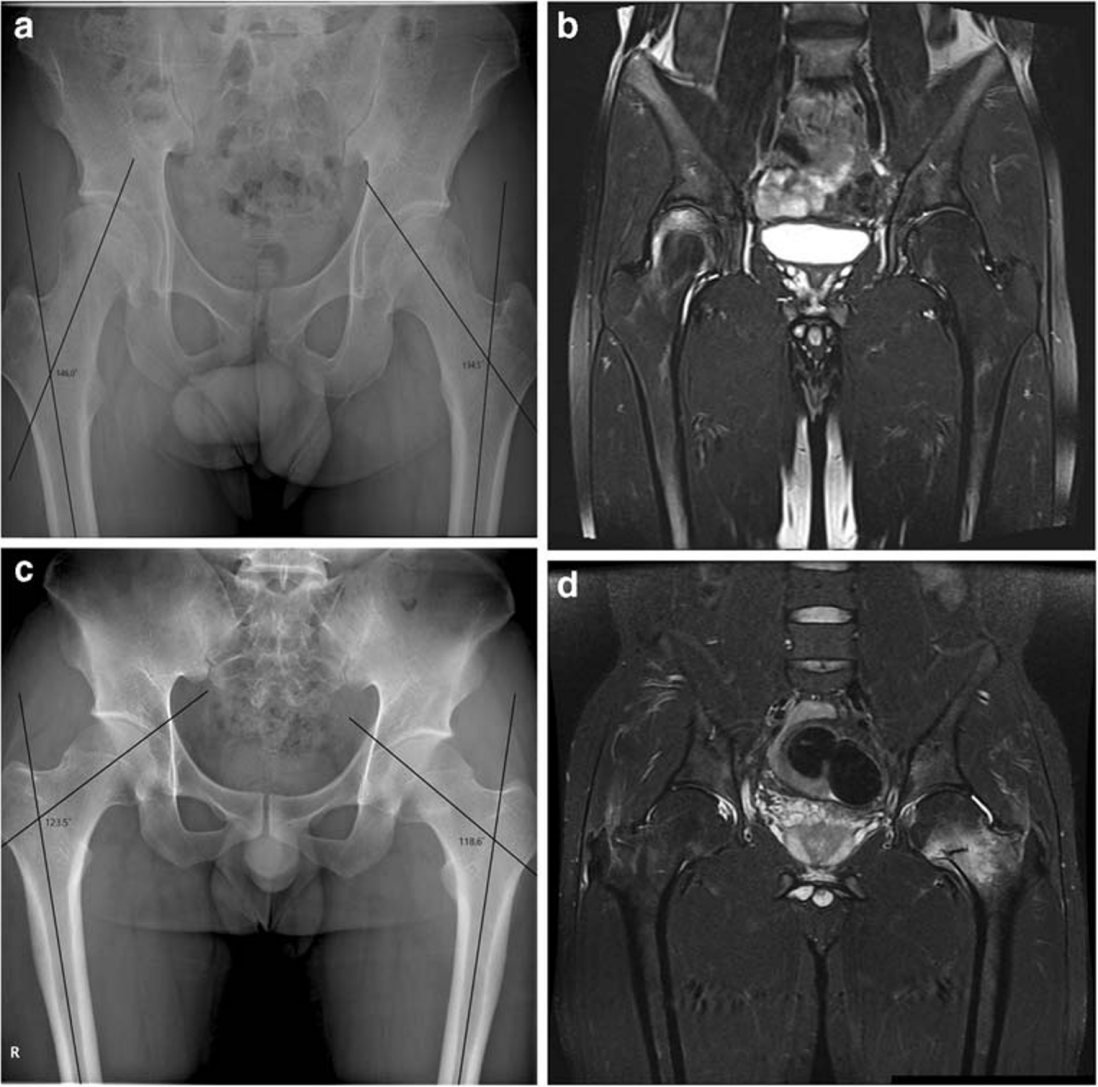
Representative cases of femoral head and neck stress fractures. In a 21-year-old man, hip anteroposterior (AP) plain radiograph (**a**) shows a FNSA of 146.0° at the right femur (coxa valga) and the coronal STIR image (**b**) shows bone marrow edema with a convex fracture line at the right femoral head, representing a subchondral stress fracture. On the contrary, in a 20-year-old man, hip AP plain radiograph (**c**) shows a FNSA of 118.6° at the left femur (coxa vara) and the coronal STIR image (**d**) shows bone marrow edema with a fracture line at the left femoral neck, representing a stress fracture

The interobserver agreement for FNSA and FNSA classification in all studied patients were 0.907 (95% confidence interval (CI) 0.833–0.949) and 0.904 (95% CI 0.851–0.957), respectively. There was a significant difference only in the FNSA and FNSA classification between the two groups. The median FNSA of group A (135.9°, IQR 129.5–138.6°) was significantly higher than that of group B (124.3°, IQR 119.5–129.0°, *p* < 0.001) (Fig. 1). Additionally, there was a significantly higher proportion of coxa valga in group A and coxa vara in group B (*p* = 0.004) (Table 1).

### Fractured versus normal femurs

**Fig. 2:**
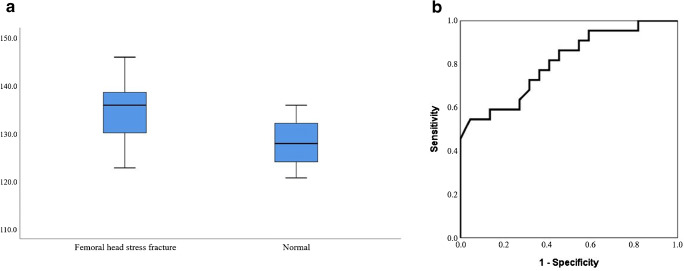
In patients with femoral head stress fractures, a box-plot (**a**) shows FNSA is significantly higher at the fractured femur (median 135.9°, range 129.9–138.6°) than that of the contralateral normal femur (median 127.9°, range 123.8–132.1°) in the same patient (*p* < 0.001). The ROC curve (**b**) of FNSA for predicting the risk of femoral head stress fracture shows the AUC of 0.807 at a cut-off value of 131.0°, with a sensitivity of 72.7% and specificity of 68.2%

Except for four patients with bilateral femoral head fractures, there were 22 patients with unilateral femoral head stress fractures, and all 11 patients with femoral neck stress fractures suffered from unilateral femoral fracture. Upon evaluation of the FNSA between the fractured femur and the contralateral normal femur in the same patients, there was a significant difference in patients with femoral head stress fractures (fractured femur, median 135.9°, IQR 129.9–138.6° vs. normal, median 127.9°, IQR 123.8–132.1°, *p* < 0.001) (Fig. 2a), but there were no significant differences in patients with femoral neck stress fractures (Table 2).

**Table 2:** Evaluation of FNSA between the fractured femur and the contralateral normal femur in the same patient: 22 patients with unilateral femoral head stress fractures and 11 patients with unilateral femoral neck stress fractures

	Head fracture femur (*n* = 22)	Contralateral normal (*n* = 22)	*p* value
FNSA (°)*	135.9 (129.9–138.6)	127.9 (123.8–132.1)	< 0.001
FNSA classification, *n* (%)			< 0.001
Normal	10 (45.5)	21 (95.5)	
Coxa vara	0 (0.0)	0 (0.0)	
Coxa valga	12 (54.5)	1 (4.5)	
	Neck fractured femur (*n* = 11)	Contralateral normal (*n* = 11)	*p* value
FNSA (°)	122.8 (121.6–127.9)	126.6 (123.5–127.7)	0.264
FNSA classification, *n* (%)			0.138
Normal	9 (81.8)	11 (100.0)	
Coxa vara	2 (18.2)	0 (0.0)	
Coxa valga	0 (0.0)	0 (0.0)	

*FNSA*, femoral neck shaft angle

*Results of continuous values are expressed as the median with interquartile range (25–75%)

ROC analysis revealed that a FNSA greater than 131.0° represented a potential cut-off value for the prediction of the risk of a femoral head stress fracture, with a sensitivity of 72.7% and a specificity of 68.2% (AUC 0.807, 95% CI 0.680–0.934, *p* < 0.001) (Fig. 2b).

## Discussion

In our study, the influence of FNSA in relation to femoral stress fracture was evaluated in military recruits controlled for most of the known risk factors for femoral stress fracture, with the exception of training risks [3–6]. This study demonstrated that FNSA was significantly associated with the occurrence and location of the femoral stress fracture. The FNSA was significantly higher in patients with femoral head stress fractures than those with femoral neck stress fractures. The proportion of coxa valga was significantly higher in patients with femoral head stress fractures, while that of coxa vara was significantly higher in those with femoral neck stress fractures. Although the FNSA values were comparable between the fractured femurs and the contralateral normal femurs in patients with femoral neck fractures (*n* = 11), a difference in FNSA was observed between the fractured femurs and the contralateral normal femurs in patients with femoral head fractures (*n* = 22). Furthermore, ROC analysis determined that a FNSA ≥ 131.0° was a potential risk factor for femoral head stress fracture.

The underlying biomechanism of femoral neck shaft anatomy in stress fractures remains unclear. Previous studies presented the effect of FNSA on knee joint osteoarthritis or femoral neck fracture, but no prior studies have been conducted on the potential correlation between the location of the stress fracture and FNSA [10, 11, 15]. The main two opposing forces across the hip joint are the body weight and the force induced by the abductor muscles. Deviation from the normal femoral neck shaft anatomy could place excessive force on the hip joint and on the femoral head and neck. In patients with coxa valga, the tip of the greater trochanter is positioned below the center of the femoral head, the abductor muscle is lengthened, and the abductor lever arm is decreased. This leads to the increase of the stress on the femoral head but decrease of the stress on the femoral neck. However, in patients with coxa vara, the tip of the greater trochanter is positioned above the femoral head center, the abductor muscle is shortened, and the abductor lever arm is increased. This results in increased stress on the femoral neck. Therefore, the coxa valga and coxa vara could serve as intrinsic risk factors for femoral head and femoral neck stress fractures, respectively [2, 16, 17]. Our results are consistent with the biomechanism of femoral neck shaft anatomy affecting the femur according to the FNSA. Furthermore, the results of the ROC analysis showed that a FNSA of greater than 131.0 ° could increase the risk of a femoral head stress fracture.

There was a significantly higher FNSA and a higher proportion of coxa valga in the fractured femur compared with the contralateral normal femur in patients with femoral head stress fractures. However, there was no significant difference in FNSA between the fractured femur and the contralateral normal femur in patients with femoral neck stress fractures, while previous investigations revealed the association between coxa vara and femoral neck stress fractures [18–21]. In our study, only 11 patients suffered from femoral neck stress fractures while 26 patients suffered from femoral head stress fractures. The small number of patients with femoral neck stress fractures may explain the discrepancy between our results and those reported in previous studies.

Our study has some limitations. First, there may be selection bias because our study was designed as retrospective study and all the included patients had bone marrow edema with noticeable fracture lines. However, sometimes it may be difficult to differentiate transient bone marrow edema syndrome or avascular necrosis from stress fracture if there was only bone marrow edema without fracture lines. Therefore, it may accomplish accurate diagnosis. Second, there was no comparison with the normal patient group, who have no lesions on the bilateral femur at MRI. However, the FNSA of each femur in the same patient who had unilateral femoral stress fractures was compared, and it could provide better control of the variables than matching and analyzing different patients. Third, the size of the study population was relatively small that may account for inconsistencies between previous study results. Furthermore, our study was designed within a particular population of military recruits. However, this current study can be considered a pilot study to look into whether FNSA is an intrinsic risk factor for femoral head and neck stress fracture among young men with activity duty. If the relationship between coxa valga and femoral head stress fracture is identified with further research in a general population, it can help predict fractures among people who undergo training considering these risk factors, which in turn can assist in choosing the intensity of training. In addition, it can also help with faster diagnosis and treatment when pain occurs and even prevent stress fracture with early diagnosis and treatment if the condition is congenital. Therefore, further study with large sample size in a general population is needed.

In conclusion, the FNSA was associated with the location of the femoral stress fracture. Coxa valga with a FNSA of greater than 135° was related to femoral head stress fractures, while coxa vara with a FNSA smaller than 120° was related to femoral neck stress fractures. Furthermore, patients with a FNSA greater than 131° could be at risk of developing femoral head stress fractures.
